# Monthly Summary

**Published:** 1871-05

**Authors:** 


### MONTHLY SUMMARY.

*Carbolic Acid Preparations.*—Mr. T. A. Baldwin, in a paper read before the British Pharmaceutical Conference, gives the following as advisable proportions in the use of carbolic acid:

As a rule it is better to dissolve the crystallized carbolic acid in the proportion of one part by weight of the acid to six of glycerine (carbolate of glycerine). In this state it can be diluted equally indefinitely.

In general, a dose of carbolic acid is one grain in an ounce of water.

As a gargle, 1 or 2 grains to an ounce af water.

As an injection 1 grain to 4 ounces of water.

As a lotion, 15 grains to an ounce of water.

As an ointment, 30 grains to an ounce of benzoated lard.

As a liniment, 1 grain to 20 ounces of olive oil.

As a plaster, 1 part of carbolic acid to 3 of shellac.

The crystallized carbolic acid to be used as a caustic.

The carbolate of glycerine, as above should be used in 1 or 2 drop doses.

Antiseptic oil for abscesses, 1 part of acid to 4 of boiled linseed oil.

Antiseptic putty, 6 spoonsful of the antiseptic oil mixed with common whiting.

Aqueous solution of carbolic acid is 1 part acid to 40 of water (1 ounce of acid to a quart of hot water, well agitated and filtered.)

Sick rooms to disinfect: place a portion of the dissolved acid in a porcelain dish, and float it in a larger vessel of hot water.

Disinfecting purposes generally: 1 pound of *crystals* to 6 gallons of water. *Fluid*, 1 part to 80 of water. *Powder*, 1 ounce of crystals with 4 pounds of slaked lime.

For drains: take 1 pound of the fluid carbolic acid to 5 gallons of warm water.

Tooth-ache is often cured with 1 drop of carbolate of glycerine; and diarrhœa arrested in half an hour with 2 drops.

In all case of parasitic life, it is advisable to commence with very dilute carbolate of glycerine.—*Chemist and Druggist.*

*The Oil of Peppermint as an Anæsthetic*—Dr. I. J. O'Brien writes to the *Druggists' Circular*:

I see by your February number, that a correspondent of the *Lancet*, while in China, discovered the fact that the natives of that antediluvian region, when suffering from facial neuralgia or tic douloureux, applied with success the oil of peppermint as an alleviative or antidote.

Without journeying to a section of the earth so remote from this division of the globe in pursuit of anagogetical authority, I became aware of the fact several years since, in the course of my practice, that the oil of peppermint possessed palliative and anæsthetic properties eminently befitting it as a valuable adjunct to my medicinal cabinet of neuralgic curatives.

Alternating creosote as a styptic with the oil of pepermint as a sedative, I have been remarkably successful in the treatment of abscess of the maxillary sinus—alveolar abscess and necrosis of the alveoli—after removing the cause, generally ulcerated teeth or fangs, and, as a consequence, necrosed bone.

In such cases I have always adopted the positive of the antiphlogistic treatment—dispersed inflammation with the astringent heat of the creosote, and dispelled all painful consequences through the anæsthetical agency of the oil of peppermint.

---

*California Qnicksilver.*—The total annual supply of quicksilver from California is not far from 50,000 flasks, or about 3,000,000 pounds. This is used in metallurgy, and in manufacturing and art processes. The largest quantity is used by the gold miners in the amalgamating process at the various mines. A considerable amount is used by manufacturing chemists in preparing calomel, "blue pill," mercurial ointment, and various mercurial salts and plasters. The Chinese make from quicksilver that beautiful pigment "vermillion" which is so largely employed by painters in all parts of the world. It is singular that the Chinese are able to prepare a chemical compound from quicksilver which is superior to, and which commands a higher price than the same salt produced in Europe and the United States, where the arts are carried to the highest perfection. English and American vermillion, as found in the market, is far inferior in brilliancy and quality to that of the Chinese.—*Am. Artisan.*

*Mixing Plaster for Impressions.—Treating Discoloured Teeth.*—H. G. Kenneth, L.D.S. says: I have been in the laboratories of many of my *confreres*, and have never seen any of them mix their plaster of paris for impressions as I do, and I flatter myself that I get the best models I have ever seen, not a bubble or a flaw. For plaster impressions of the mouth my plan of mixing is the very best, and will cause the plaster to set immediately. I simply put it and the water in a wedgwood mortar, and grind them rapidly until sufficiently incorporated. I think we save much annoyance and loss of time by making use of such little hints. I have tried many things for bleaching discoloured teeth; have succeeded with some; failed with mostly all. Some are too caustic, others too easily deliquesced and their moral effects neutralized.

In all cases of discoloration which I can have under my control, I thoroughly remove every particle of decay, dead pulp, &c., and take chloride of lime 1 part; oxy-chloride of zinc 1 part, mix as usual with the fluid of the latter, and fill the cavity, renewing several times in the course of a month. The chloride of lime must be fresh and have been kept close corked. I was indebted to a friend a long time ago for this simple method of treatment. It has served my purpose very nicely several times.—*Canada Journal of Dental Science.*

---

*To Polish Marble, etc.*—Marble of any kind, alabaster, any hard stone, or glass, may be repolished by rubbing it with a linen cloth dressed with oxide of tin—sold under the name of putty powder. For this purpose a couple or more folds of linen should be fastened tight over a piece of wood, flat or otherwise, according to the size of the stone. To repolish a mantel piece it should first be perfectly cleaned. This is best done by making a paste of lime, soda, and water, well wetting the marble, and applying the paste. Then let it remain for a day or so keeping it moist during the interval. When this paste has been removed the polishing may begin. Chips in the marble should be rubbed out first with emery and water. At every stage of polishing the linen and putty powder must be kept constantly wet. Glass such as jewellers' show counter cases, which become scratched, may be polished in the same way.—*Druggists Circular.*

*Hydrate of Chloral.*—It appears to be generally admitted now that the use of the newly discovered hydrate of chloral, unless by the advice of a competent physician, is liable to lead to seriously injurious results; but it does not follow that the drug may not be of great benefit as a remedial agent to skilful hands. An opium eater writes to the *Chicago Tribune* that for many years he had made every possible effort to free himself from the habit› efforts that were seconded by all the aids that medical science could give, but that they had all failed from one cause, the total inability to sleep after leaving off opium. In the hydrate of chloral he found the desired relief. Until he had completely abandoned the opium he did not meddle with it; even then he used it merely to induce sleep, taking about thirty grains at the usual hour of repose. In twenty minutes after taking it he invariably slept, and in the morning awoke thoroughly refreshed. As the time passed and the effect of the opium became more and more eliminated from his system, he took less of the chloral until his cure was complete. It is not to be understood that the use of chloral destroys the taste for opium, by any means; but the great difficulty in the way of opium eaters who have sought to abandon the habit has been, that after having had the resolution to discontinue its use, the sleeplessness which was certain to ensue became so unendurable that the victim has been compelled to fall back upon his enemy for relief. To opium eaters who really have a fixed determination to escape from their degrading slavery, the hydrate of chloral, administered under the supervision of a judicious medical man, in connection with other treatment such as any competent physician would prescribe promisei to be of inestimable value.—*Journal of Materia Medica.*

---

*Death from Chloroform.*—Dr. Hughes Bennett stated, at a late meeting of the British Association, that "he knew of one very sad case that had happened at Edinburgh. A young and beautiful lady, daughter of a barrister, in perfect health, went to a dentist's house one morning, and had a tooth extracted. Five minutes afterwards she was dead. That was only one of many similar cases that had occurred, but had never been published."—*British Medical Journal.*

*Bleaching Beeswax.*—Mr. L.G. Olmstead, of New York, gives the following statement of a simple and convenient method of bleaching beeswax, that he saw practised in Italy: "The yellow wax is first melted in a kettle, and then is dipped out in a long tin vessel that will hold two or three gallons, and which has a row of small holes, about the diameter of a knitting-needle, in the bottom. The vessel is fixed over a cylinder of wood two feet in length and fifteen inches in diameter, which is made to revolve like a grindstone, in one end of a trough of water, two and one half feet in width, ten to fifteen feet in length, and one foot in depth. As the melted wax falls in small streams on this wet revolving cylinder, it flattens out into a thin ribbon and floats off towards the other end of the trough of water. It is then dipped out with a skimmer—that may be constructed of osier twigs—spread on a table with a top made of small willow rods, covered with a clean white cloth, and then exposed in this way to the sun until bleached."—*Druggists' Circular.*

---

*Death from Inhalation of Ether.*—At last a veritable case of directly fatal result from ether inhalation comes to light. It occurred in Boston, in the person of a man who had received a bullet wound in the knee, and who was etherized for the purpose of amputation. He suddenly ceased to breathe during the operation. In nearly every instance of death hitherto imputed to ether, hours, if not days, have elapsed before the fatal result. But the present case is different, resembling in this respect the action of chloroform. The particulars are given in the *Boston Medical and Surgical Journal.*

---

*Cancrum Oris.*—John Burton, M.D., Georgia, Ind., gives the following recipe for this disease in the *Journal of Materia Medica:*

| | | |
|---|---|---|
| ℞ Cupri Sulphas | ʒ | ss. |
| Pulv. Cinchona | ʒ | i. |
| Aqua | ℥ | i. M. |

Apply with a Camel's hair brush, three times a day.

I have tried this treatment for twenty years, never having it fail in my hands.

